# Habitat-specific responses of leaf traits to soil water conditions in species from a novel alpine swamp meadow community

**DOI:** 10.1093/conphys/cov046

**Published:** 2015-11-12

**Authors:** Honglin Li, Adrienne B Nicotra, Danghui Xu, Guozhen Du

**Affiliations:** af1 State Key Laboratory of Grassland and Agro-Ecosystems, School of Life Sciences, Lanzhou University, Lanzhou 730000, China; af2 Research School of Biology, Australian National University, Canberra, ACT 0200, Australia

**Keywords:** Alpine swamp meadow, climate change, gas exchange, habitat specific, leaf morphology, water availability

## Abstract

Species originally from alpine wetland and alpine meadow communities now coexist in a novel ‘alpine swamp meadow’ community as a consequence of wetland drying in the eastern Tibetan Plateau. Considering the projected increase in the fluctuation of water supply from precipitation during the growing season in this area in the future, it is important to investigate the responses of the species that make up this new community to soil water availability. Using a transplant experimental design, we compared the response of leaf traits and growth to different water conditions for species grouped according to their original habitat of wetland or meadow. Twelve perennial herbaceous species, which form an alpine swamp meadow community in Maqu County in the eastern Tibetan Plateau, were used in this study and subjected to two water treatments, namely waterlogged and dry-down. Overall, significant differences in leaf production in response to soil water availability were found for these two groups, indicating strongly different effects of water availability on their growth. Furthermore, the meadow group had lower specific leaf area, leaf area and relative leaf water content, but thicker leaves than those of the wetland group, indicating significant habitat-specific differences in leaf morphology. Regarding physiological traits, the wetland group had significantly higher photosynthetic rates in inundated conditions, whereas for the meadow group the photosynthetic rate was greatest in cyclically dry conditions. Likewise, a similar pattern was observed for stomatal conductance; however, both groups achieved higher instantaneous water use efficiency during the dry-down treatment. The results of this study indicate that the composition of the alpine swamp meadow could be sensitive to changes in precipitation and might be changed substantially by future declines in water supply, as predicted by global climate change models for this region. This potential for compositional change of the community should be considered when management and conservation decisions are made.

## Teaser

The physiological and morphological responses of plants to different water conditions in a newly formed alpine swamp meadow community in the eastern Tibetan Plateau were examined. Results showed significant habitat-specific responses and thus suggested that the alpine swamp meadow will be vulnerable to further alteration of water conditions under future climate change.

## Introduction

Wetland plant communities, whose habitats and associated species are highly dependent on hydrological conditions, are likely to be influenced by climate change, especially changes in precipitation (Dawson *et al*., 2003; Acreman *et al*., 2009). The changes in function and species composition of wetland that result from drying out and water shortage have become a problem in the protection and conservation of wetlands in many places (Dugan, 1992; Erwin, 2009; Xiang *et al*., 2009).

Zoige wetland, located in eastern part of the Qinghai-Tibet Plateau, is the largest alpine wetland in China and plays a critical role as a water resource for the Yellow River and for conservation of many plant and animal species (Xiang *et al*., 2009). However, overgrazing and misuse in conjunction with global warming during recent decades have led to severe ecosystem change (Zhang *et al*., 2011). In particular, there has been a significant trend of aridification in most wetland areas (Qiu *et al*., 2009; Xiang *et al*., 2009). With the altered soil water availability, a novel ‘alpine swamp meadow’ community has emerged, which is composed of species typical of both the alpine wetland and the alpine meadow (Xiang *et al*., 2009; Ma *et al*., 2011). Thus, the soil water conditions in the swamp meadow are likely to play a key role in shaping its species composition and structure. Furthermore, the Qinghai-Tibet Plateau has been described as extremely vulnerable to global climate change (Wang and French, 1994; Thompson *et al*., 2000), which is predicted also to influence the local precipitation conditions. More extreme weather events, including both heavy rain and drought, will be more common under changed climate (Knapp *et al*., 2008; Smith, 2011); this could have pronounced impacts on the local vegetation, particularly on the novel swamp meadow community.

The species composition of a given plant community is assembled through habitat-filtering processes (Weiher and Keddy, 1999; Cornwell *et al*., 2006). Keedy (1992) suggested that the habitat selects for a set of species that are somewhat equivalent functionally. As a result of human activity and climate change in recent decades, many novel environments have been created (Mahlstein *et al*., 2011, 2013), because of which novel plant communities are emerging, where species composition is mixed from adjacent habitats. The response of species from these communities to environmental factors should be partly derived from and determined by their original habitat (Kudo and Hirao, 2006; Song *et al*., 2013); for example, their traits may provide a fitness advantage in environments that are similar to the original local environmental conditions (local adaptation; see Kawecki and Ebert, 2004). Alternatively, the plants may be highly plastic and thus show convergent traits; for example, adjusting their traits to accommodate new habitat conditions (Nicotra *et al*., 2010). To our knowledge, few studies have examined the habitat-specific characteristics or plastic response of species that originated elsewhere and now occur in a newly formed habitat, such as the alpine swamp meadow.

Soil water conditions have long been seen as one of the most important environmental factors for plant establishment, growth and distribution in many vegetation types (Bray, 1997; Chaves *et al*., 2002; Otsus and Zobel, 2004; García-Sánchez *et al*., 2007; Flexas et al., 2009; Li *et al*., 2011). In the alpine area, for instance, studies conducted where periods of drought are frequent during the growing season show that water availability limits plant growth and ecosystem productivity (Scott and Billings, 1964; Walker *et al*., 1994; Fisk *et al*., 1998). Leaf morphological traits, such as specific leaf area (SLA) and leaf thickness, play an important role in plant growth, and they are often influenced by environmental factors (Cunningham *et al*., 1999; Niinements, 2001). Gas-exchange properties are the most fundamental of plant physiological processes and are also affected by environmental conditions (Wang and Gao, 2001; Wang and Yuan, 2001; Almeida *et al*., 2014). For example, photosynthetic capacity strongly affects the growth and dry-matter production of plants (Hirasawa and Hsiao, 1999; Chen *et al*., 2005; El-Sharkawy, 2011; Catoni and Gratani, 2014) and is greatly affected by soil water conditions (Kimenov *et al*., 1989; Anderson *et al*., 1995; Sanhueza et al., 2015). Thus, assessing plant performance in response to water conditions by measuring morphological traits and photosynthetic capacity in different water conditions is a useful approach for understanding the contrasting biology of co-occuring plants in the swamp meadow community.

Using a transplant experimental design, we simulated the two contrasting water conditions that are likely to prevail as consequence of future climate change in the alpine swamp meadow: a mosaic of waterlogged soil and drier soils that are subjected to repeated dry-down. We assessed the responses of leaf morphological (e.g. SLA, leaf thickness) and physiological traits (e.g. gas exchange) to soil water content. The objectives of this study were to determine whether there are habitat-specific differences in trait means and response to water conditions indicative of local adaptation to conditions similar to historic water regimens and to describe the pattern of the response of these species to different water conditions. *A priori*, we expected a significant but different response of these groups of species to a change in soil water availability reflecting the habitat specificity and/or local adaptation of the species. As a result, we predicted that the plant community of alpine swamp meadow should be unstable and largely influenced by future water regimen, especially precipitation. Understanding the patterns of change in precipitation in future climate change scenarios and reducing other disturbances (such as overgrazing) should be considered in management and conservation decisions for this novel community.

## Materials and methods

### Study species and area

Twelve common species (from eight families) were selected from the alpine swamp meadow plant community (Table 1); six are typical wetland species, whereas the others are usually abundant in alpine meadows (Wu *et al*., 1994). All of these species are perennial herbaceous plants, in which only the below-ground structures survive through winter. In total, these species account for >50% of total above-ground biomass (55.43 ± 4.32%) and coverage (58.46 ± 3.81%) of the swamp meadow community (H. Li, A. B. Nicotra, D. Xu and G. Du, unpublished work).

**Table 1:** The 12 perennial species (eight families) used in experiments

Group	Species	Family
Wetland	*Caltha palustris*	Ranunculaceae
	*Carex kansuensis*	Cyperaceae
	*Nardostachys chinensis*	Valerianaceae
	*Potentilla anserina*	Rosaceae
	*Rumex aquaticus*	Polygonaceae
	*Sanguisorba filiformis*	Rosaceae
Meadow	*Anemone coelestina* var. *linearis linearis*	Ranunculaceae
	*Elymus nutans*	Poaceae
	*Kobresia capilifolia*	Cyperaceae
	*Oxytropis kansuensis*	Fabaceae
	*Saussurea hieracioides*	Asteraceae
	*Trollius farreri*	Ranunculaceae

The research was conducted at the Field Station of Alpine Meadow and Wetland Ecosystems of Lanzhou University (Maqu branch), in the eastern Qinghai-Tibet Plateau. The site is situated at latitude 33°39′N, longitude 101°53′E, and the average elevation is 3660 m. This site has a typical plateau continental climate with short, cool summers and long, cold winters (Rui *et al*., 2012). The annual average temperature is 2.2°C and annual precipitation is 672 mm, falling primarily as rain between the months of June and August (Chu *et al*., 2008, Niu *et al*., 2014). The growing season is short, with July the season of peak plant growth. Leaves become senescent from mid-August.

### Experimental design

In mid-May 2012, 12 species were transplanted from the field into individual plastic pots containing soil from the swamp meadow site. The diameter of the pots was 20 cm at the top and 18 cm at the bottom, and the height was 14 cm. Forty plants per species, each of which had only one or two small leaves emerged (enough for successful identification of the species) were planted, one per pot. Individuals were selected at a consistent size and, presumably, age for each species. The plastic pots were filled with soil from the same site [soil was carefully sieved and mixed before use, with total nitrogen 0.81 ± 0.03% and total phosphorus 0.87 ± 0.09 mg g^−1^, which were measured by taking soil cores and analysing with an elemental analyser (FIAstar 5000 Flow Injection Analyzer, FOSS, Denmark)] and arranged in a plastic-covered metal shelter to prevent the pots from receiving natural rainfall. The transmittance of the plastic roof was 85%, measured by a PAR light meter (Decagon Devices, Pullman, WA, USA). A plastic sheet below prevented the plants from accessing ground moisture and kept the irrigation water from seeping into ground soil.

Two weeks after the plants been transplanted, the four least healthy plants per species were excluded and the remaining 36 plants per species were randomly assigned to two water regimens, either the waterlogged treatment (W) or the repeated dry-down treatment (D), to simulate the potential water conditions as consequence of climate change. The 18 pots per treatment were randomly divided into one of three sub-plots containing six pots per species. Pots of a given sub-plot were placed on plastic sheets, which were laid on the ground with the edges fixed a little higher than ground level. During the experimental time, the waterlogged treatment was irrigated with standing water to about 5 cm deep around the pots at all times. The repeated dry-down treatment for a given sub-plot was adjacent to the waterlogged treatment, and plants were given 300 ml water (∼80% of soil field capability) and re-watered every 4 days, at which point the soil water content was about 40–50% of field capability. Plants were observed to ensure that the treatment did not cause wilting during the experimental period (from June to August).

### Gas exchange, leaf morphology and growth measurements

Both leaf gas exchange and morphological traits were measured for one fully expanded, newly developed, healthy leaf on each of six plants (randomly selected from 18 pots) for each species in each treatment in mid-July, 6 weeks after water treatment was imposed. These leaves had all developed and expanded after the treatment was imposed (personal observation). Leaf gas exchange was conducted between 10.00 and 12.30 h during a sunny, clear day (third day after watering for dry-down treatment) in mid-July with a GSF-3000 portable photosynthesis system (GFS-3000; Heinz Walz, Germany). The measurements were taken at 1800 µmol m^−2^ s^−1^, which equates to the average light intensity of sunlight during the measurement (measured by the ambient light sensor of GFS-3000), thus ensuring that light was not limiting. The CO_2_ concentration was ∼340 ppm (reflecting the measured ambient concentration on site), and relative humidity was between 50 and 65%. The instantaneous water use efficiency (WUEi) was calculated as photosynthetic rate (*A*_max_)/transpiration rate (*E*).

For leaf morphology, one fully expanded, newly developed, healthy leaf from each of the six randomly selected pots was first weighed, then scanned (EPSON 1670; Seiko Epson Corporation, Japan), then dried at 80°C for 48 h and reweighed. The area of each leaf was calculated using ImageJ (Rasband, 2005). The specific leaf area (SLA = area/dry weight, in square metres per gram), leaf thickness (measured with digital callipers to the nearest millimetre), leaf area (LA, in square centimetres) and relative leaf water content [RLWC; (fresh weight-dry weight)/fresh weight, expressed as a percentage] were calculated for each leaf.

At the end of the experiment (mid-August), the total number of leaves (including those that were not fully expanded) generated during the water treatment were counted for each plant as a proxy for growth.

### Statistical analysis

The effect of water conditions and species' original habitat (wetland or meadow) on leaf morphology, gas-exchange traits and growth were analysed using two-way ANOVA (type III sum of squares, with treatment and group as main effects, and species as random factors, see Supplementary material Fig. S1 for the responses of individual species to the water conditions). The differences between treatments or between groups for all measured traits were analysed using ANOVA at *P* = 0.05. Data that violated the ANOVA assumptions of normality of variance were log-transformed. All statistical analyses were performed in SPSS 16.0 for Windows (SPSS, Chicago, IL, USA).

## Results

**Figure 1:**
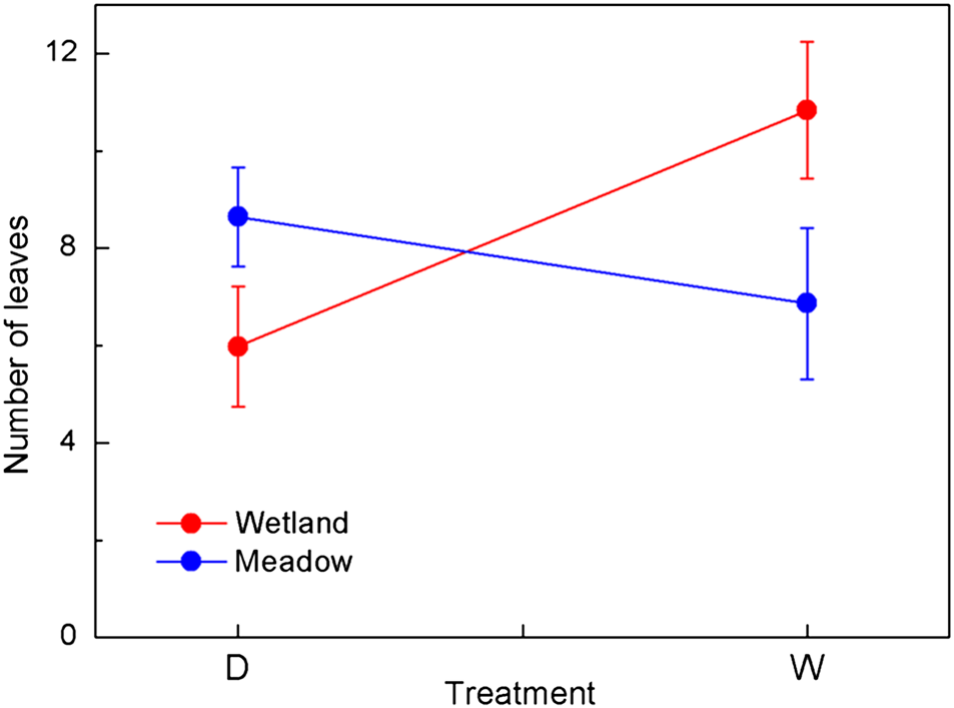
Number of leaves (means ± 1 SE, a proxy for growth) that emerged after treatment was applied for wetland and meadow species in different water treatments. Red line indicates wetland species and blue line meadow species. Abbreviations: D, dry-down treatment; and W, waterlogged treatment.

The growth (indicated by leaf production) of the two groups showed strong differences in response to water treatment (Table 2A and Fig. 1). There were significantly more leaves generated in the waterlogged than the dry-down treatment for the wetland group (*P* < 0.001), whereas more leaves were generated in the dry-down than the waterlogged conditions for the meadow group (Fig. 1; not significant, *P* = 0.107).

**Table 2:** Significance values from ANOVA of measured traits for two groups grown in waterlogged and dry-down treatments

		Treatment		Group		Treatment × group	
	Traits	*F*	*P*-value	*F*	*P*-value	*F*	*P*-value
A	Number of leaves	4.589	0.034*	0.823	0.366	21.273	<0.001*
B	SLA (m^2^ g^−1^)	2.99	0.088	22.67	<0.001*	2.56	0.114
	Leaf thickness (mm)	0.93	0.337	9.92	0.031*	1.58	0.212
	LA (cm^2^)	1.35	0.249	15.93	0.024*	2.13	0.149
	RLWC (%)	1.33	0.253	9.06	0.002*	1.47	0.462
C	*A*_max_ (µmol CO_2_ m^−2^ s^−1^)	8.39	0.005*	0.09	0.758	7.56	0.009*
	*g*_s_ (mmol m^−2^ s^−1^)	7.96	0.007*	0.02	0.885	8.01	0.007*
	WUEi [µmol CO_2_ (mmol H_2_O)^−1^]	10.54	0.002*	0.52	0.474	0.75	0.390

A, the growth traits; B, leaf morphological traits; C, leaf physiological traits. Degree of freedom for treatment and group = 1 and the denominator d.f. = 120. Abbreviations and terms: *A*_max_, net photosynthesis rate; *g*_s_, the stomatal conductance for CO_2_; LA, leaf area; leaf thickness, the thickness of a single leaf in the middle; number of leaves, the total number emerged after the treatment applied, is a proxy for growth; RLWC, relative leaf water content; SLA, specific leaf area; and WUEi, instantaneous water use efficiency.

*Indicates significant effect of treatment or their interaction (*P*-value < 0.05).

**Figure 2:**
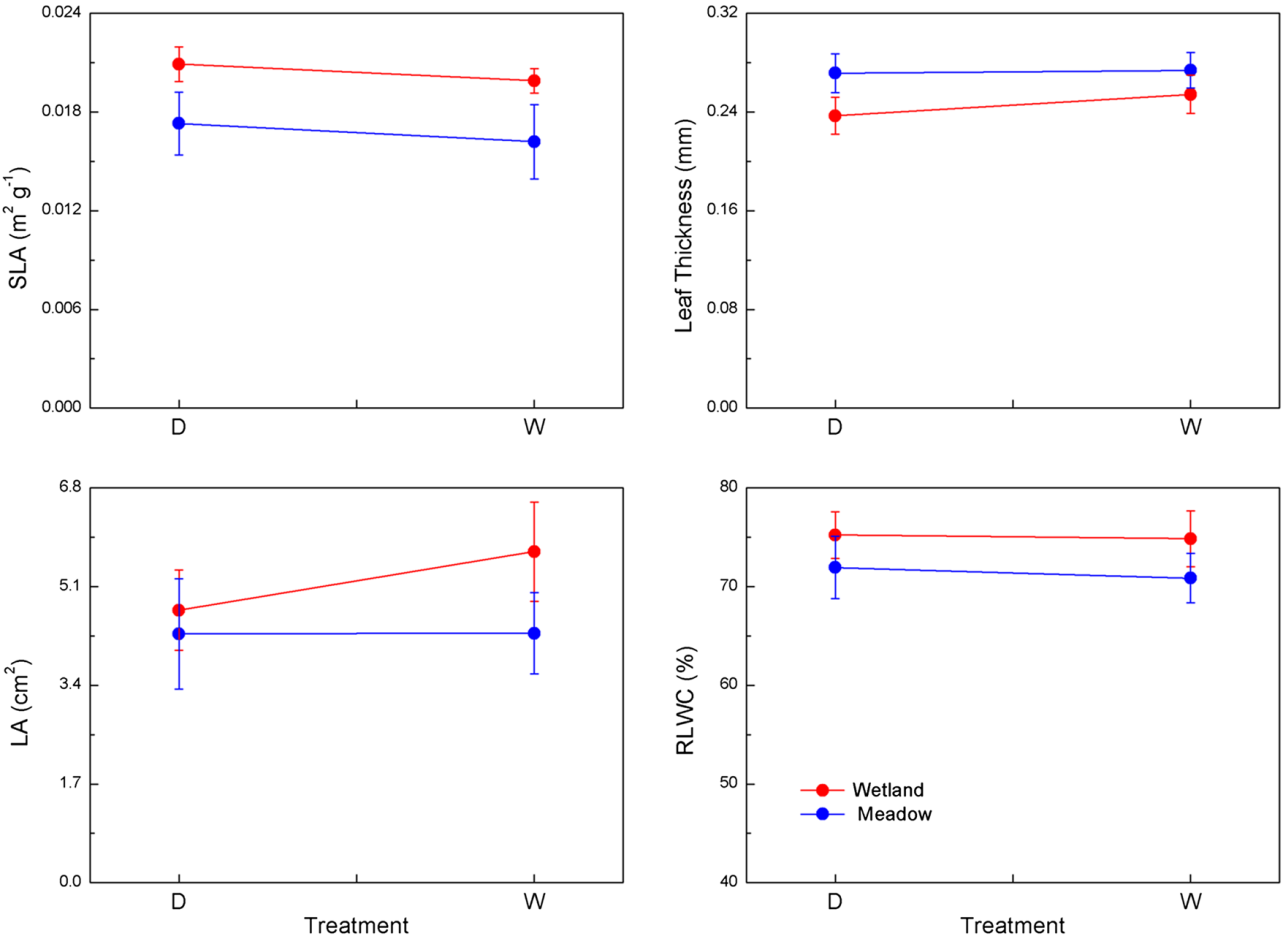
Leaf morphological traits (means ± 1 SE) of the two groups (wetland group, red lines; meadow group, blue lines) in different water conditions. Abbreviations: D, dry-down; LA, leaf area; leaf thickness, the thickness of a single leaf in the middle; RLWC, relative leaf water content; SLA, specific leaf area; and W, waterlogged.

There were significant differences between groups for leaf morphological traits, but neither group showed significant effects of water treatment (Table 2B and Fig. 2). The meadow group had significantly lower SLA, LA and RLWC but greater leaf thickness compared with the wetland group (Fig. 2; *P* < 0.05).

**Figure 3:**
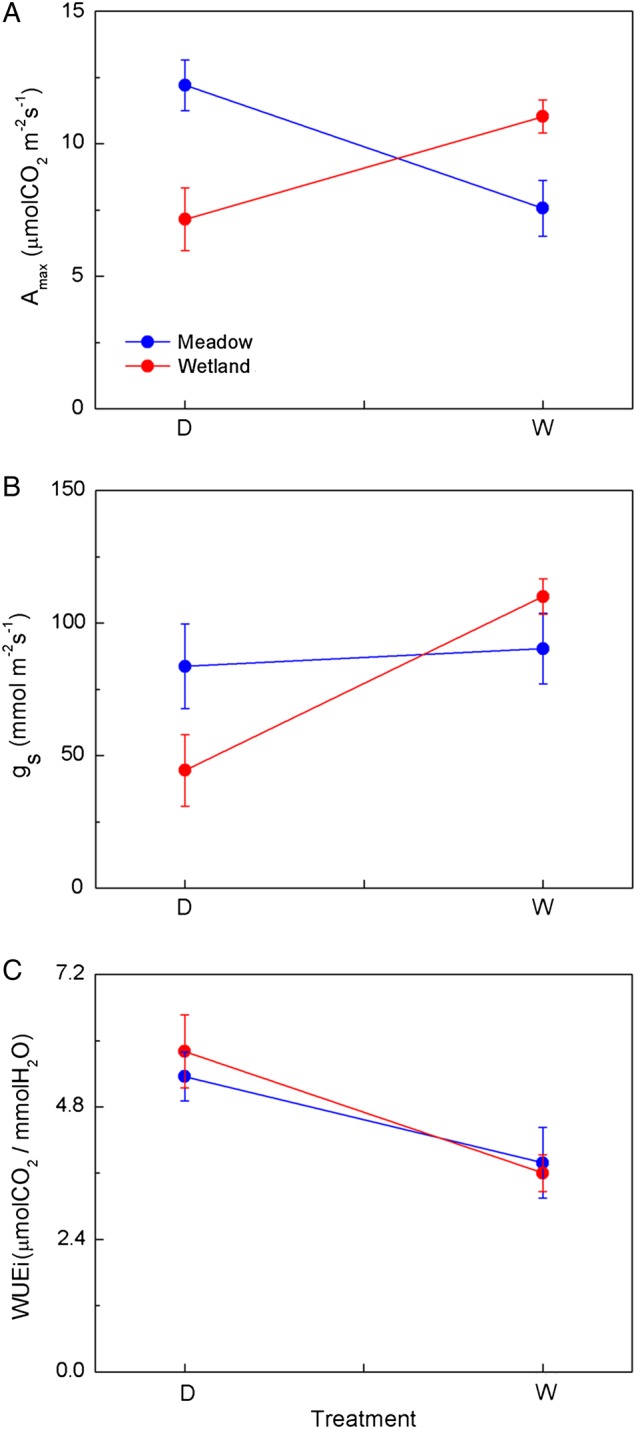
Mean value (±1 SE) of the net photosynthesis rate (*A*_max_; **A**), stomatal conductance (*g*_s_; **B**) and instantaneous water use efficiency (WUEi; **C**) of wetland and meadow species in different water conditions. Red lines indicate wetland species and blue lines meadow species. Abbreviations: D, dry-down treatment; and W, waterlogged treatment.

In contrast, both water treatment and the interaction between water treatment and species groups significantly affected gas-exchange traits (Table 2C and Fig. 3). The wetland group had significantly higher *A*_max_ when grown in waterlogged than in dry-down conditions (*P* < 0.05), whereas the rankings were reversed for the meadow group (Fig. 3A; *P* < 0.05). Likewise, the stomatal conductance (*g*_s_) of wetland group was significantly higher in waterlogged than dry-down conditions (Fig. 3B; *P* < 0.001), whereas the meadow group showed no significant effect of growth water conditions on *g*_s_ (*P* = 0.535). In dry-down conditions, the *A*_max_ and *g*_s_ of meadow group species was significantly higher than that of the wetland group (*P* < 0.05), whereas the *A*_max_ of the wetland group was significantly higher than that of the meadow group in waterlogged conditions (*P* = 0.005). There was no significant difference between groups for *g*_s_ in waterlogged conditions (*P* = 0.362). The WUEi of both groups was higher in dry-down conditions, and there was not a significant difference between species groups (Fig. 3C; *P* > 0.05).

## Discussion

Using a controlled experiment, we tested the responses of plant species in a newly formed ‘novel’ swamp meadow community to different soil water treatments; the significant but distinctive responses of the two groups of species to changes in soil water availability followed our predictions. Given that the swamp meadow community was formed only recently, following the drying of wetland (Xiang *et al*., 2009; Ma *et al*., 2011), the species that emerged in this site could either reflect species from each of the original communities that were highly plastic and thus would show convergent traits in the novel community or, alternatively, could have maintained their original habitat-specific characteristics (local adaptation) and thus show stronger group differences than treatment effects. Song *et al*. (2013) found that the responses of germination and seedling establishment to soil water conditions are habitat specific in alpine plants on the Tibetan Plateau. Similar signals of habitat specificity are also evident in our study. We found that overall, wetland species performed best (as indicated by leaf growth) in flooded conditions and meadow species best in periodic drought conditions. We also found that gas-exchange traits reflected significant effects of both water treatment and habitat of origin. In contrast, we did not find a significant effect of water conditions on morphological traits for either group of species, although the species groups differed markedly in these traits. This difference in the pattern of response could reflect that ours was a relatively modest water stress (compared with others; see Kramer, 1969) that might not have been strong enough to affect morphological traits (e.g. Nicolás *et al*., 2005), that we did not impose the stress for long enough to elicit a morphological shift or, alternatively, the result may suggest that morphology is a ‘more expensive’ shift to achieve and that in this system the species have evolved high levels of plasticity in physiological traits that underlie canalization in morphological ones. Given that significant effects of water treatment on growth were observed, we find the final option most plausible.

Our finding that the wetland group performed better in waterlogged conditions, whereas the meadow group performed better in dry-down conditions is consistent with the habitat specificity, potentially reflecting local adaptation of these groups. A recent study that examined the responses of dominant grass species in a mesic tallgrass prairie to drought has reported contrasting strategies that different species employed to respond to water shortage (Hoover *et al*., 2014). The results from our study also indicate the sensitivity of these alpine species to water availability, especially for the wetland species (Fenner and Freeman, 2011). In addition, our results highlight the important role of soil water availability in controlling this novel plant community and the high vulnerability of the current community structure to future changes in precipitation regimen, which is expected to become increasingly variable according to climate change projections for the Tibetan Plateau (Liu *et al*., 2006; You *et al*., 2008). On the one hand, the fluctuating water conditions may effectively create additional niches that species can occupy, thus promoting coexistence and biodiversity, but on the other hand, if cycles of water availability occur on longer time scales they could result in widespread mortality in either wetland or meadow species. Resulting areas of bare ground could lead to exacerbated water loss, erosion and further degradation of water-holding capacity of the swamp meadow.

Species from both wetland and meadow exhibited higher WUEi in dry-down than waterlogged conditions. The underlying mechanism, however, reflected differences in the physiology and morphology of these two groups of species. In the waterlogged conditions, the wetland species had significantly higher rates of carbon uptake compared with the dry-down conditions, whereas in the meadow species the pattern was reversed. The higher WUEi in the dry-down treatment for wetland species was associated with a steep decrease of *g*_s_, whereas for meadow species increased WUEi resulted from increased *A*_max_, seemingly somewhat uncoupled from *g*_s_ (see Farquhar and Sharkey, 1982). Likewise, the combination of higher SLA and LA and lower leaf thickness in the wetland group is typical of species found in mesic environments (Niinemets, 2001; Nicotra *et al*., 2007). These results suggest a degree of local adaptation (Kawecki and Ebert, 2004).

Theory suggests that the greater the difference in functional traits between species, the more likely there will be a strong difference in response to limiting factors (Adler *et al*., 2013). Here, we demonstrated significant differences in leaf traits and growth response between the wetland and meadow in response to water availability. The results suggest that the community composition of the alpine swamp meadow in the eastern Qinghai-Tibet Plateau is likely to remain highly sensitive to changes in water conditions and could be substantially influenced by future water supply; thus, it is unstable. At this point, we cannot make a firm prediction about which way it will go; therefore, further research is needed in order to inform management and conservation decisions adequately. Wetland aridification is accelerating in the Tibetan Plateau as well as on a global scale (Dugan, 1992; Xiang *et al*., 2009). Our study makes a substantive contribution to understanding of species and community dynamics of the novel swamp meadow in this time of significant global climate change (Liu *et al*., 2006; Knapp *et al*., 2008; You *et al*., 2008; Smith, 2011). Our results may assist with conservation, management and plant community restoration actions; for example, management to reduce disturbances that may affect soil water availability (e.g. overgrazing, drainage and peat exploitation) could improve stability of the community.

## Supplementary material

Supplementary material is available at *Conservation Physiology* online.

## Funding

This work was supported by the National Natural Science Foundation of China (grant no. 41171214) and the Chinese Scholarship Council.

## Supplementary Material

Supplementary Data

## References

[COV046C2] Acreman MC, Blake JR, Booker DJ, Harding RJ, Reynard N, Mountford JO, Stratford CJ (2009) A simple framework for evaluating regional wetland ecohydrological response to climate change with case studies from Great Britain. Ecohydrology 2: 1–17.

[COV046C3] Adler PB, Fajardo A, Kleinhesselink AR, Kraft NJB (2013) Trait-based tests of coexistence mechanisms. Ecol Lett 16: 1294–1306. 23910482 10.1111/ele.12157

[COV046C4] Almeida A-AF, Gomes FP, Araujo RP, Santos RC, Valle RR (2014) Leaf gas exchange in species of the *Theobroma* genus. Photosynthetica 52: 16–21.

[COV046C5] Anderson JE, Nowwk RS, Rasmuson KE, Toft NL (1995) Gas exchange and resource-use efficiency of *Leymus cinereus* (Poaceae): diurnal and seasonal responses to naturally declining soil moisture. Am J Bot 82: 699–780.

[COV046C7] Bray EA (1997) Plant responses to water deficit. Trends Plant Sci 2: 48–54.

[COV046C8] Catoni R, Gratani L (2014) Variations in leaf respiration and photosynthesis ratio in response to air temperature and water availability among Mediterranean evergreen species. J Arid Environ 102: 82–88.

[COV046C9] Chaves MM, Pereira JS, Maroco J, Rodrigues ML, Ricardo CP, Osório ML, Carvalho I, Faria T, Pinheiro C (2002) How plants cope with water stress in the ﬁeld. Photosynthesis and growth. Ann Bot 89: 1–10. 10.1093/aob/mcf105 PMC4233809 12102516

[COV046C10] Chen SP, Bai YF, Lin GH, Liang Y, Han XG (2005) Effects of grazing on photosynthetic characteristics of major steppe species in the Xilin River Basin, Inner Mongolia, China. Photosynthetica 43: 559–565.

[COV046C11] Chu CJ, Maestre FT, Xiao S, Weiner J, Wang YS, Duan ZH, Wang G (2008) Balance between facilitation and resource competition determines biomass–density relationships in plant populations. Ecol Lett 11: 1189–1197. 18684118 10.1111/j.1461-0248.2008.01228.x

[COV046C12] Cornwell WK, Schwilk DW, Ackerly DD (2006) A trait-based test for habitat filtering: convex hull volume. Ecology 87: 1465–1471. 16869422 10.1890/0012-9658(2006)87[1465:attfhf]2.0.co;2

[COV046C13] Cunningham SA, Summerhayes B, Westoby M (1999) Evolutionary divergences in leaf structure and chemistry, comparing rainfall and soil nutrient gradients. Ecol Monogr 69: 569–588.

[COV046C14] Dawson TP, Berry PM, Kampa E (2003) Climate change impacts on freshwater wetland habitats. J Nat Conserv 11: 25–30.

[COV046C15] Dugan P (1992) Wetlands in Danger. Mitchell Beazley London Ltd, London.

[COV046C16] El-Sharkawy MA (2011) Overview: early history of crop growth and photosynthesis modeling. BioSystems 103: 205–211. 20826195 10.1016/j.biosystems.2010.08.004

[COV046C17] Erwin KL (2009) Wetlands and global climate change: the role of wetland restoration in a changing world. Wetlands Ecol Manage 17: 71–84.

[COV046C18] Farquhar GD, Sharkey TD (1982) Stomatal conductance and photosynthesis. Annu Rev Plant Physiol 33: 317–345.

[COV046C19] Fenner N, Freeman C (2011) Drought-induced carbon loss in peatlands. Nat Geosci 4: 895–900.

[COV046C20] Fisk MC, Schmidt SK, Seastedt TR (1998) Topographic patterns of above- and below-ground production and nitrogen cycling in alpine tundra. Ecology 79: 2253–2266.

[COV046C21] Flexas J, Barón M, Bota J, Ducruet JM, Gallé A, Galmés J, Jiménez M, Pou A, Ribas-Carbó M, Sajnani C et al (2009) Photosynthesis limitations during water stress acclimation and recovery in the drought-adapted *Vitis* hybrid Richter-110 (*V. berlandieri*×*V. rupestris*). J Exp Bot 60: 2361–2377. 19351904 10.1093/jxb/erp069

[COV046C22] García-Sánchez F, Syvertsen JP, Gimeno V, Botía P, Perez-Perez JG (2007) Responses to flooding and drought stress by two citrus rootstock seedlings with different water-use efficiency. Physiol Plantarum 130: 532–542.

[COV046C23] Hirasawa T, Hsiao TC (1999) Some characteristics of reduced leaf photosynthesis at midday in maize growing in the ﬁeld. Field Crops Res 62: 53–62.

[COV046C24] Hoover DL, Knapp AK, Smith MD (2014) Contrasting sensitivities of two dominant C4 grasses to heat waves and drought. Plant Ecol 215: 721–731.

[COV046C25] Kawecki TJ, Ebert D (2004) Conceptual issues in local adaptation. Ecol Lett 7: 1225–1241.

[COV046C26] Keddy PA (1992) Assembly and response rules: two goals for predictive community ecology. J Veg Sci 3: 157–164.

[COV046C27] Kimenov GP, Markovska YM, Tsonev TD (1989) Photosynthesis and transpiration of *Haberlea rhodopensis* FRIV. in dependence on water deficit. Photosynthetica 23: 368–371.

[COV046C28] Knapp AK, Beier C, Briske DD, Classen AT, Luo Y, Reichstein M, Smith MD, Smith SD, Bell JE, Fay PA et al (2008) Consequences of more extreme precipitation regimes for terrestrial ecosystems. BioScience 58: 811–821.

[COV046C30] Kramer PJ (1969) Plant and Soil Water Relationships: a Modern Synthesis. MaGraw-Hill, New York.

[COV046C31] Kudo G, Hirao AS (2006) Habitat-speciﬁc responses in the ﬂowering phenology and seed set of alpine plants to climate variation: implications for global change impacts. Popul Ecol 48: 49–58.

[COV046C32] Li F, Li YZ, Qin HG, Xie YH (2011) Plant distribution can be reﬂected by the different growth and morphological responses to water level and shade in two emergent macrophyte seedlings in the Sanjiang Plain. Aquat Ecol 45: 89–97.

[COV046C33] Liu XD, Yin ZY, Shao XM, Qin NS (2006) Temporal trends and variability of daily maximum and minimum, extreme temperature events, and growing season length over the eastern and central Tibetan Plateau during 1961–2003. J Geophys Res 111: D19109.

[COV046C34] Ma MJ, Zhou XH, Du GZ (2011) Soil seed bank dynamics in alpine wetland succession on the Tibetan Plateau. Plant Soil 346: 19–28.

[COV046C35] Mahlstein I, Knutti R, Solomon S, Portmann RW (2011) Early onset of significant local warming in low latitude countries. Environ Res Lett 6, 034009.

[COV046C36] Mahlstein I, Daniel JS, Solomon S (2013) Pace of shifts in climate regions increases with global temperature. Nat Clim Change 3: 739–743.

[COV046C37] Nicolás E, Torrecillas A, Dell'Amico J, Alarcón JJ (2005) The effect of short-term flooding on the sap flow, gas exchange and hydraulic conductivity of young apricot trees. Trees 19: 51–57. 10.1016/j.jplph.2004.05.014 15900886

[COV046C38] Nicotra AB, Hermes JP, Jones CS, Schlichting CD (2007) Geographic variation and plasticity to water and nutrients in *Pelargonium australe*. New Phytol 176: 136–149. 17803645 10.1111/j.1469-8137.2007.02157.x

[COV046C39] Nicotra AB, Atkin OK, Bonser SP, Davidson AM, Finnegan EJ, Mathesius U, van Kleunen M (2010) Plant phenotypic plasticity in a changing climate. Trends Plant Sci 15: 684–692. 20970368 10.1016/j.tplants.2010.09.008

[COV046C40] Niinemets Ü (2001) Global-scale climatic controls of leaf dry mass per area, density, and thickness in trees and shrubs. Ecology 82: 453–469.

[COV046C41] Niu KC, Choler P, Bello FD, Mirotchnick N, Du GZ, Sun SC (2014) Fertilization decreases species diversity but increases functional diversity: a three-year experiment in a Tibetan alpine meadow. Agr Ecosyst Environ 182: 106–112.

[COV046C42] Otsus M, Zobel M (2004) Moisture conditions and the presence of bryophytes determine fescue species abundance in a dry calcareous grassland. Oecologia 138: 293–299. 14593527 10.1007/s00442-003-1428-8

[COV046C43] Qiu PF, Wu N, Luo P, Wang ZY, Li MH (2009) Analysis of dynamics and driving factors of wetland landscape in Zoige, Eastern Qinghai-Tibetan Plateau. J Mountain Sci 6: 42–55.

[COV046C44] Rasband WS (2005) ImageJ. US National Institutes of Health, Bethesda, MD, USA.

[COV046C45] Rui YC, Wang YF, Chen CG, Zhou XQ, Wang SP, Xu ZH, Duan JC, Kang XM, Lu SB, Luo CY (2012) Warming and grazing increase mineralization of organic P in an alpine meadow ecosystem of Qinghai-Tibet Plateau, China. Plant Soil 357: 73–87.

[COV046C46] Sanhueza C, Bascunan-Godoy L, Turnbull MH, Corcuera LJ (2015) Response of photosynthesis and respiration to temperature under water deﬁcit in two evergreen *Nothofagus* species. Plant Species Biol 30: 163–175.

[COV046C47] Scott D, Billings WD (1964) Effects of environmental factors on standing crop and productivity of an alpine tundra. Ecol Monogr 34: 243–270.

[COV046C48] Smith MD (2011) An ecological perspective on extreme climatic events: a synthetic definition and framework to guide future research. J Ecol 99: 656–663.

[COV046C49] Song B, Stöcklin J, Gao YQ, Zhang ZQ, Yang Y, Li ZM, Sun H (2013) Habitat-speciﬁc responses of seed germination and seedling establishment to soil water condition in two *Rheum* species in the high Sino-Himalayas. Ecol Res 28: 643–651.

[COV046C50] Thompson LG, Yao T, Mosley-Thompson E, Davis ME, Henderson KA, Lin PN (2000) A high-resolution millennial record of the South Asian monsoon from Himalayan ice cores. Science 289: 1916–1919. 10988068 10.1126/science.289.5486.1916

[COV046C51] Walker MD, Webber PJ, Arnold EH, Ebert-May D (1994) Effects of interannual climate variation on aboveground phytomass in alpine vegetation. Ecology 75: 393–408.

[COV046C52] Wang B, French HM (1994) Climate controls and high-altitude permafrost, Qinghai-Xizang (Tibet) Plateau, China. Permafrost Periglac 5: 87–100.

[COV046C53] Wang RZ, Gao Q (2001) Photosynthesis, transpiration, and water use efficiency in two divergent *Leymus chinensis* populations from Northeast China. Photosynthetica 39: 123–126.

[COV046C54] Wang RZ, Yuan YQ (2001) Photosynthesis, transpiration, and water use efficiency of two *Puccinellia* species on the Songnen grassland, northeastern China. Photosynthetica 39: 283–287.

[COV046C55] Weiher E, Keddy P (1999) Assembly rules as general constraints on community composition. In Weiher E, Keddy PA, eds, Ecological Assemble Rules: Perspectives, Advances, Retreats. Cambridge University Press, Combridge, UK, pp 251–271.

[COV046C56] Wu ZY, Raven PH, Hong DY (1994) Flora of China. Science Press & Missouri Botanical Garden, Beijing, St Louis http://flora.huh.harvard.edu/china/.

[COV046C57] Xiang S, Guo RQ, Wu N, Sun SC (2009) Current status and future prospects of Zoige Marsh in Eastern Qinghai-Tibet Plateau. Ecol Eng 35: 553–562.

[COV046C58] You QL, Kang SH, Aguilar E, Yan YP (2008) Changes in daily climate extremes in the eastern and central Tibetan Plateau during 1961–2005. J Geophys Res 113: D07101.

[COV046C59] Zhang Y, Wang GX, Wang YB (2011) Changes in alpine wetland ecosystems of the Qinghai-Tibetan plateau from 1967 to 2004. Environ Monit Assess 180: 189–199. 21140209 10.1007/s10661-010-1781-0

