# Supplementary material for: Habitat-specific responses of leaf traits to soil water conditions in species from a novel alpine swamp meadow community
Source: Conserv Physiol. 2015 Nov 12;3(1):cov046. doi: 10.1093/conphys/cov046 (PMC4778432; doi:10.1093/conphys/cov046)
Supplement: Supplementary Data [file cov046supp.zip › cov046supp.docx]

**Supplemental information**


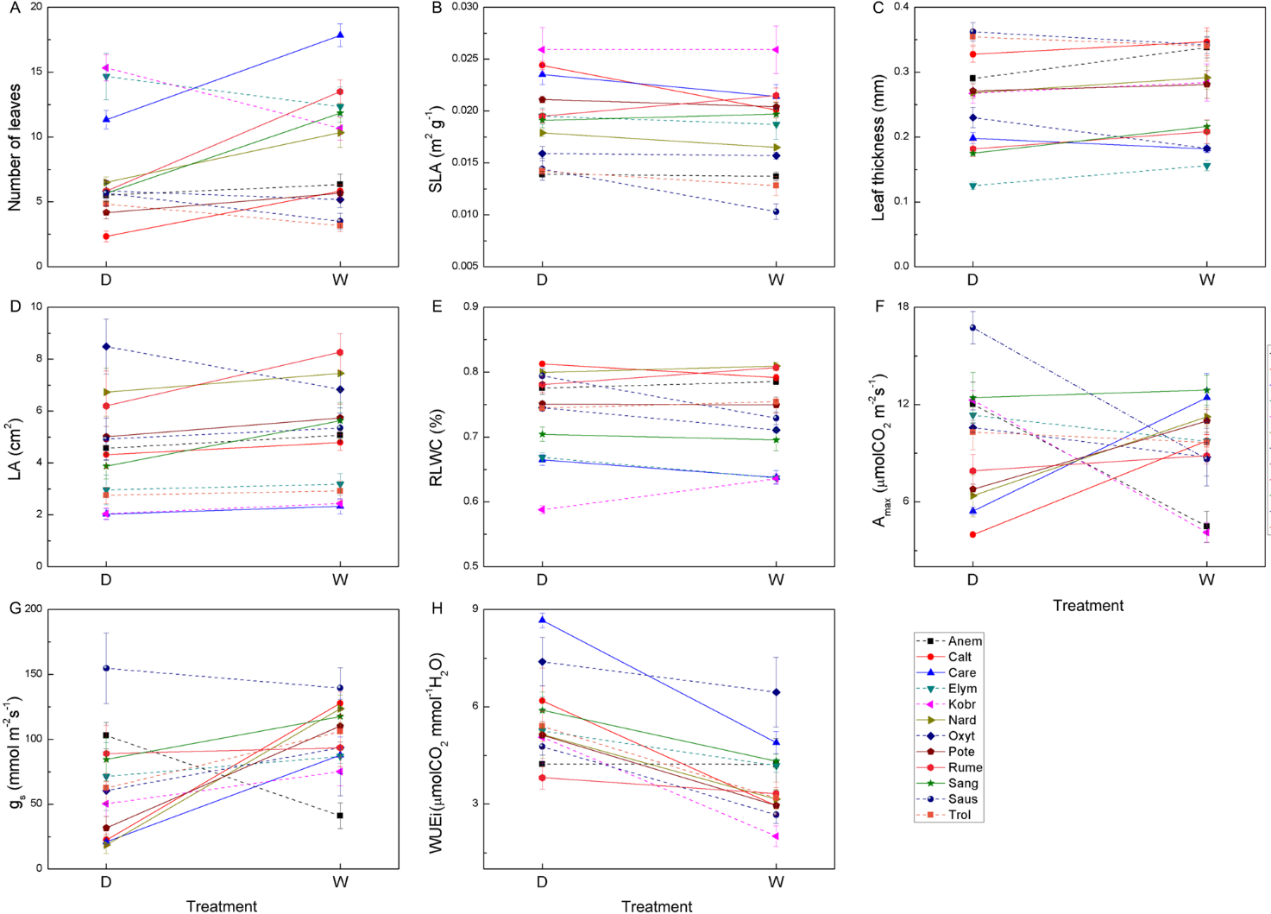


**Supplemental Fig. 1. Mean value (± 1SE) of the Number of leaves emerged after treatment was applied (A), the specific leaf area (SLA, B), the leaf thickness (C), the leaf area (LA, D), relative leaf water content (RLWC, E), the net photosynthesis rate (A_max_, F), stomatal conductance (g_s_, G) and instantaneous water use efficiency (WUEi, H) for 12 species from alpine wetland (solid lines) and alpine meadow (dotted lines). Anem, *Anemone coelestina var. linearis linearis*; Calt, *Caltha palustris*; Care, *Carex kansuensis*; Elym, *Elymus nutans*; Kobr, *Kobresia capilifolia*; Nard, *Nardostachys chinensis*; Oxyt, *Oxytropis kansuensis*; Pote, *Potentilla anserina*; Rume, *Rumex aquaticus*; Sang, *Sanguisorba filiformis*; Saus, *Saussurea hieracioides*; Trol, *Trollius farreri*.**
